# The Role of Magnetic Resonance Imaging in the Management of Esophageal Cancer

**DOI:** 10.3390/cancers14051141

**Published:** 2022-02-23

**Authors:** Anna Pellat, Anthony Dohan, Philippe Soyer, Julie Veziant, Romain Coriat, Maximilien Barret

**Affiliations:** 1 Department of Gastroenterology and Digestive Oncology, Hôpital Cochin, AP-HP, 27 rue du Faubourg Saint Jacques, 75014 Paris, France; anna.pellat@aphp.fr (A.P.); romain.coriat@aphp.fr (R.C.); 2 Université de Paris, 75006 Paris, France; anthony.dohan@aphp.fr (A.D.); philippe.soyer@aphp.fr (P.S.); julie.veziant@aphp.fr (J.V.); 3 Department of Radiology, Hôpital Cochin, AP-HP, 27 rue du Faubourg Saint Jacques, 75014 Paris, France; 4 Department of Digestive Surgery, Hôpital Cochin, AP-HP, 27 rue du Faubourg Saint Jacques, 75014 Paris, France

**Keywords:** esophageal cancer, magnetic resonance imaging, staging

## Abstract

**Simple Summary:**

Esophageal cancer (EC) is the eighth most frequent cancer worldwide, with a poor prognosis. Current imaging modalities for staging and follow-up mainly include computed tomography (CT), positron emission tomography (PET)/CT, and endoscopic ultrasound. Magnetic resonance imaging (MRI), which is a non-irradiating and non-invasive modality, can provide identification of the esophageal wall and esophagogastric junction. MRI has shown encouraging capabilities in regional and local staging of EC as well as in the assessment of treatment response to therapy. Technical refinements in MRI technique and sequences overtime have contributed to its increasing diagnostic performance as well as its generalizability. MRI could become a routine imaging technique for EC management in the future, alone or in combination with other modalities.

**Abstract:**

Esophageal cancer (EC) is the eighth more frequent cancer worldwide, with a poor prognosis. Initial staging is critical to decide on the best individual treatment approach. Current modalities for the assessment of EC are irradiating techniques, such as computed tomography (CT) and positron emission tomography/CT, or invasive techniques, such as digestive endoscopy and endoscopic ultrasound. Magnetic resonance imaging (MRI) is a non-invasive and non-irradiating imaging technique that provides high degrees of soft tissue contrast, with good depiction of the esophageal wall and the esophagogastric junction. Various sequences of MRI have shown good performance in initial tumor and lymph node staging in EC. Diffusion-weighted MRI has also demonstrated capabilities in the evaluation of tumor response to chemoradiotherapy. To date, there is not enough data to consider whole body MRI as a routine investigation for the detection of initial metastases or for prediction of distant recurrence. This narrative review summarizes the current knowledge on MRI for the management of EC.

## 1. Introduction

According to the global cancer observatory, esophageal cancer (EC) is currently the eighth most common cancer worldwide and the sixth most common cause of death by cancer (https://gco.iarc.fr/, accessed on 17 December 2021). About 600,000 cases were diagnosed in 2020, with more than 540,000 deaths. Both incidence and mortality rates vary greatly across countries [1]. Until recently, surgery and the combination of chemoradiotherapy have been the main treatments for EC in the localized setting. The recent CheckMate 577 trial has shown improved disease-free survival for patients treated with nivolumab, an immune checkpoint inhibitor, in the adjuvant setting of operated EC after neoadjuvant chemoradiotherapy [2]. In the metastatic setting, chemotherapy and immune checkpoint inhibitors are the main available treatments [3,4].

Upon diagnosis, clinical and radiological staging is crucial to propose the optimal treatment strategy to patients. Several imaging techniques, including computed tomography (CT), positron emission tomography (PET)/CT, endoscopic ultrasound (EUS), as well as esophagogastroduodenoscopy, are recommended by European guidelines in the management of EC [5,6]. A preoperative CT classification has been proposed by Bosset et al. (cTNM) [7]. The TNM staging system, provided by the American Joint Committee on Cancer (AJCC) and the International Union Against Cancer (UICC), is used for pathologic tumor classification of the disease [8]. Contrary to other modalities, magnetic resonance imaging (MRI) is a non-irradiating and non-invasive technique. It also provides excellent soft-tissue contrast. It is not currently a routine examination for the management of EC because of its relatively low availability and its technical limitations. Nevertheless, it seems a promising technique for tumoral staging, delineation of target volumes before chemoradiotherapy, response to treatment and prediction of recurrence. Improvement of MRI modalities over the years, and the development of a larger choice of sequences, have contributed to enhancing MRI performance in EC.

The purpose of this narrative review was to sum up the current evidence on the role of MRI in the management of EC.

## 2. MRI Modalities for the Esophagus

### 2.1. Major Technical Developments in MRI of the Esophagus Overtime

Measurements and description of the normal esophagus in the sagittal view were first assessed in 78 patients using electrocardiogram (ECG)-gated MRI. In 2004, Manabe et al. compared T1-weighted ultrafast gradient echo (TFE) MRI and T1-weighted fast field echo (FFE) MRI in 20 healthy volunteers, with the aim to delineate the esophageal passage under dynamic conditions [9]: The fast field echo images proved superior in terms of signal to noise ratio and overall quality [9]. The use of an external surface coil and cardiac gating with T2-weighted fast spin-echo (FSE) sequences was shown in 2006 to further improve the signal of the quality of esophageal MRI, and the speed of acquisition [10]. Later, an ex vivo study using high field MRI with a similar protocol helped precisely define the MRI anatomy of the posterior mediastinum [11]. Similarly, in a study on 33 operated patients with EC, preoperative high-resolution T2-weighted FSE sequence provided detailed images and comparison with histology findings showed good correlation between the degree of esophageal wall infiltration and pathological T-staging [12]. Furthermore, ultra-high-resolution T2-weighted MRI at 7.0-T provided clear definition of the esophageal wall and excellent accuracy for the T staging [13]. In 2017, pre-treatment motion-triggered MRI was used to improve the description of the periesophageal tissue [14].

Dynamic MRI of the esophagus, using oral administration of various contrast agents, has been proposed to assess the esophageal peristalsis on sagittal images [15]. Gadopentetate dimeglumine mixed with barium [16], ferric ammonium citrate-cellulose paste [17], buttermilk spiked with gadolinium chelate [15], concentrated pineapple juice mixed with potato starch [18] were tested with satisfactory results, allowing to describe the esophagus in the sagittal plane over a mean of 16 cm, and define normal values for the esophageal transit time [19,20]. Noticeably, the latter intraluminal agent provided a similar image quality to those obtained with paramagnetic contrast agents.

### 2.2. How We Do It in Our Center

In our center, we obtain T1-weighted images in the coronal and axial planes before and after intravenous administration of a gadolinium chelate; then T2-weighted single shot spine echo sequences in the axial and coronal plane, and dynamic kinematic acquisition of steady-states sequences are obtained in the oblique plane, parallel to the esophagogastric junction. Patients are asked to swallow water through a straw during the dynamic acquisition phase. This is repeated several times in order to visualize the esophageal contractions. The sequences parameters are presented in the Table 1. The correct placement of sagittal oblique kinematic sequences is of paramount importance to visualize the esophagus and the esophagogastric junction. Indeed, the presence of the heart in the acquisition box will lead to motion artifacts. Morphological signs are analyzed on T2 weighed images and steady state cine sequences.

**Figure 1:**
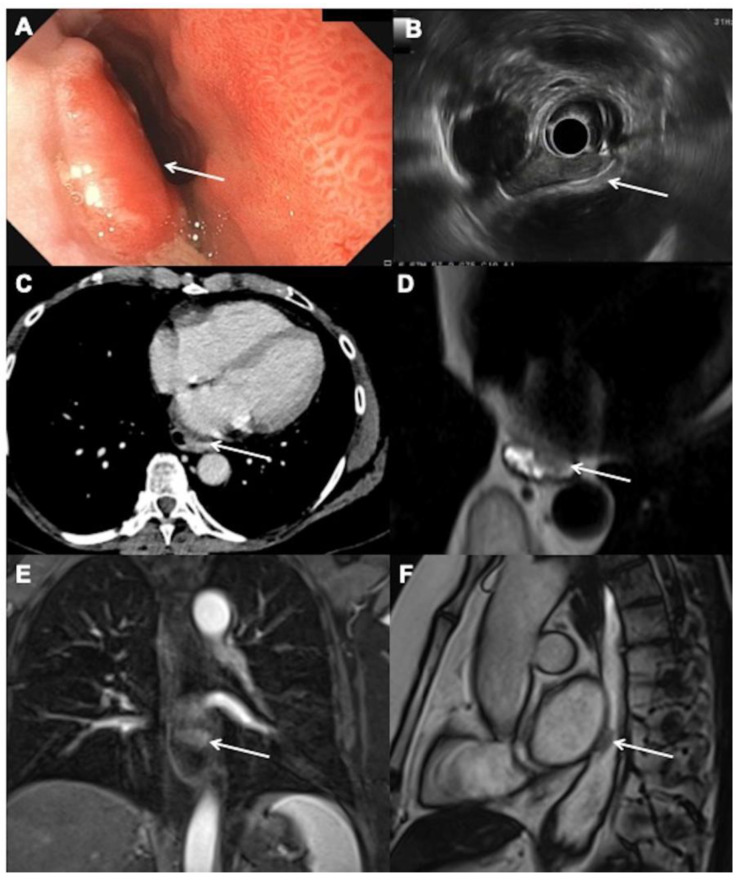
Initial staging for a 66-year-old woman with T2N0M0 esophageal adenocarcinoma: (**A**) endoscopic view of an elevated neoplastic lesion arising on a Barrett’s esophagus on the left posterior side of the esophagus, 34 cm from the dental arch (arrow). (**B**) Endoscopic ultrasound showing the hypoechoic, well-limited neoplastic lesion in close contact with the muscularis propria without regional lymph nodes (arrow). (**C**) Contrast-enhanced computed tomography image obtained at 70 s after intravenous administration of iodinated contrast material and without oral contract material in the axial plane shows irregular esophagus (arrow) but no definite lesion. (**D**) Axial T2-weighted single shot magnetic resonance (MR) images. after oral administration of water show a low signal irregular anterior lesion (arrow). (**E**) Coronal contrast-enhanced T1 weighted images confirm the lesion that demonstrates an early contrast uptake (arrow). (**F**) Sagittal steady-state MR images confirm the low signal lesion (arrow) and allow to precisely see its location.

**Figure 2:**
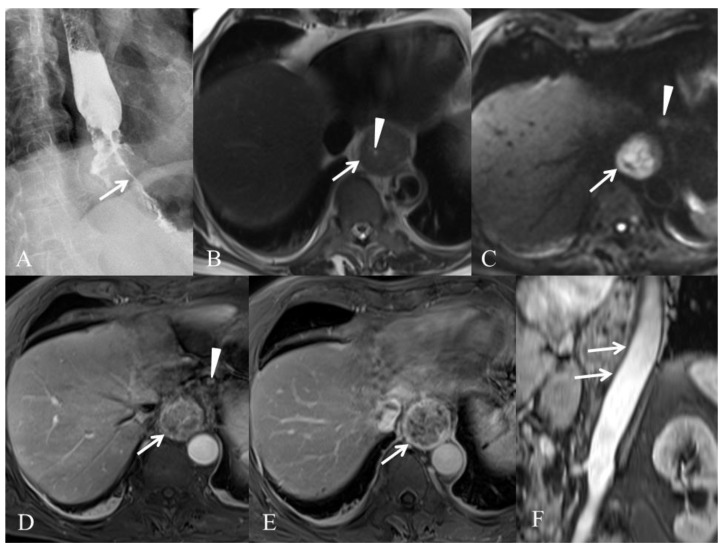
Initial staging for a 77-year-old man with T3N1M0 esophageal adenocarcinoma. (**A**) Esophagram shows esophageal tumor of the lower third of the esophagus responsible for marked luminal narrowing (arrow). (**B**) T2-weighted HASTE MR image in the axial plane shows esophageal tumor (arrow) with luminal narrowing (arrowhead). (**C**) Diffusion-weighted MR image in the axial plane obtained with high b value (b = 800 s/mm^2^) shows restricted diffusion (arrow) consistent with malignant esophageal tumor. Additional hyperintense lymph node is present (arrowhead). (**D**) T1-weighted VIBE image in the axial plane obtained 30 s after intravenous administration of a gadolinium-based contrast agent (gadoterate meglumine, Dotarem^®^, Guerbet, Villepinte, France) shows heterogeneous esophageal tumor (arrow) and enhancing lymph node (arrowhead). (**E**) T1-weighted VIBE image in the axial plane obtained 60 s after intravenous administration of a gadolinium-based contrast agent (gadoterate meglumine, Dotarem^®^, Guerbet) shows that the tumor is well delineated without spreading outside the adventitia (arrow). (**F**) MR angiography image in the oblique plane shows intact interface (arrows) between esophageal tumor and aorta.

We present in Figure 1 and Figure 2 the results of different modalities used for initial staging of EC in two different patients.

## 3. MRI and Esophageal Cancer (EC) Diagnosis and Staging

### 3.1. Initial Tumor (T) Staging

One of the major challenges in EC is the evaluation of local staging in order to choose the best treatment approach. For low stage EC, the optimal treatment between upfront surgery or administration of neoadjuvant therapy is still unclear and will be influenced by robust staging.

Tumor (T) staging is often done with EUS. In 2008, a meta-analysis on preoperative EC including 49 studies found pooled sensitivities ranging from 81.6 to 92.4% for differentiation of different T-stages, with a better performance in advanced (T4) disease [21]. Pooled specificities were 99.4% and 97.4% for T1 and T4 cancers, respectively [21]. In a more recent meta-analysis on preoperative ESCC, the overall accuracy of EUS for T-staging was 79% (95% CI: 88–94) [19]. EUS is superior to CT for evaluation of T-staging since CT cannot distinguish the different histological layers [20,22,23]. CT can be reliable when it comes to determining resectability by excluding high T-stages tumors [24,25]. ^18^F-fluorodeoxyglucose (^18^F-FDG) PET/CT has a limited role for T-staging due to its low spatial resolution and is mainly useful for the diagnosis of distant metastases [24,26]. Although the local and regional staging accuracy of EUS is greater than those of CT and PET [27], EUS is invasive and operator dependent, and sometimes limited by tumor stenosis [28].

With this in mind, MRI seems a promising tool for the evaluation of T-staging in EC. Various in vitro studies found that MRI could clearly describe the different layers of the esophageal wall and had a high diagnostic accuracy for evaluating mural invasion [29,30]. Similar results were found in the ex-vivo setting with both high-resolution T2-weighted and diffusion-weighted MRI (DWI) [31,32,33], as well as in the in vivo setting [12,13]. Improvement of MRI modalities over the years has contributed in increasing the performance of MRI diagnosis and staging in EC [34,35]. Nevertheless, the heterogeneity of MRI modalities and sequences used in different studies limits the generalizability of currently available results. The T2-weighted FSE technique evaluated in 39 patients showed high accuracy in differentiating between T2 and T3 disease but with a tendency to overstage T1 tumors [12]. One trial evaluating the influence of two different volumetric interpolated breath-hold sequences (VIBE) on T-staging in EC, found that contrast-enhanced free-breathing radial VIBE was superior to breath-hold Cartesian VIBE, especially for T1 and T2 stage EC [36]. MR esophagography with water swallowing was evaluated in 30 patients with thoracic EC and 10 healthy volunteers [37]. By comparison with conventional MRI, it showed better results for assessing the tumor’s length and exact localization, but lower accuracy for T-staging [38]. T2*-weighted imaging had good accuracy for the evaluation of T-staging in patients with ESCC, except for the differentiation between T0 and T1-stage tumors [39]. In a study by Wu et al., gross tumor volume (GTV) assessed on T2-weighted MRI, contrast-enhanced T1-weighted and DWI in 60 patients with ESCC was associated with T-stage and the presence of lymph node metastases [40]. They also reported that GTV diagnosed on contrast-enhanced T1-weighted imaging better predicted T-stage [40]. Furthermore, one work suggested that whole-tumor histogram analysis of some pharmacokinetic parameters from dynamic contrast-enhanced (DCE)-MRI might be able to predict T-stage in ESCC [41]. In addition, MR angiography of the thorax in the same session provides very useful information about the invasiveness of the cancer in vessels, vascular anomalies, including arterial and venous status.

A recently published meta-analysis on 20 trials addressed the issue of MRI diagnostic performance for EC, including the question of precise T-staging [35]. With 11 trials published between 2009 and 2019 addressing the question of differentiation between T0 and T1 or more advanced disease, MRI had a pooled sensitivity of 92% (95% CI: 82–96) and specificity of 67% (95% CI: 51–81). The administration of neoadjuvant chemoradiotherapy did not significantly impact these results [35]. With 10 studies evaluating differentiation between T2 or lower disease and T3 or higher disease, MRI had a pooled sensitivity of 86% (95% CI: 76–92) and a specificity of 86% (95% CI; 75–93). Unfortunately, the authors did not address the specific question of the diagnostic value of MRI to differentiate between T0 or T1 tumor (amenable to endoscopic low morbidity resection) and ≥T2 tumors (requiring chemoradiotherapy and/or surgical resection). Overall, MRI had a good sensitivity for T-staging in EC.

Evidence in favor of a high accuracy for T-staging by MRI in preoperative EC is growing, even if there is heterogeneity between available studies in terms of MRI sequences, study designs and histological subtypes of EC. MRI shows good sensitivity for low T-stages and good sensitivity and specificity for higher T-stages. In the close future, MRI alone, or in combination with other modalities, will probably be used in routine clinical practice for early T-staging of EC.

### 3.2. Node (N) Staging

Upon diagnosis, regional staging with evaluation of node (N) staging is also crucial to evaluate prognosis and choose the optimal treatment approach. Not only is survival correlated with the T-stage, it is also clearly influenced by the N-stage [42]. Indeed, pathological evidence of lymph node metastases is a major prognostic factor in operated EC [37,42] with 5-year overall survival rates ranging between 70 and 92% in case of negative node involvement versus 18% to 47% for patients with positive lymph node involvement [43,44]. Furthermore, the lymph node ratio, which is the number of infiltrated lymph nodes divided by the total number of resected lymph nodes, is also an independent factor of survival for operated patients [45].

Currently, baseline regional lymph node involvement in EC is also preferably evaluated with EUS, followed by CT and ^18^F-FDG-PET/CT [24,25,26]. EUS can give access to fine needle aspiration (FNA) for histologic evaluation of regional lymph nodes (mediastinum and coeliac). In a 2008 meta-analysis, EUS showed a pooled sensitivity of 80% (95% CI: 75–84) and pooled specificity of 70% (95% CI: 65–75) for N-staging [46]. Several studies have suggested that EUS-FNA had a greater accuracy than EUS alone for N-staging [47,48]. In the meta-analysis by Puli et al., pooled sensitivity of N-staging with EUS improved from 84.7 (95% CI: 82.9–86.4) to 96.7% (95% CI: 92.4–98.9) with FNA [21]. In the meta-analysis by Van Vliet et al., both CT and ^18^F-FDG-PET/CT showed lower sensitivities for regional N-staging in EC, 50% (95% CI: 41–60) and 57% (95% CI: 43–70), respectively [46]. Regarding ^18^F-FDG-PET/CT, this could partly be explained by the difficulty in the distinction of lymph nodes adjacent to a highly avid primary tumor with a high standard uptake value.

Because of the previously described limitations of current techniques for N-staging, MRI has also been evaluated in this setting. Early studies using conventional MRI at 0.35–1.5 T without fast sequences reported sensitivities, specificities and diagnostic accuracies of 25–70%, 67–93% and 56–89% respectively [49,50,51,52]. More recent studies with similar modalities support these findings and found similar sensitivities, specificities, and accuracies of 38–62%, 68–85% and 64–77%, respectively [53,54]. Superparamagnetic iron oxide (SPIO), which is phagocytized by macrophages after intravenous administration, has enhanced the value of MRI in detecting lymph node metastases. Indeed, metastatic lymph nodes show a marked reduction in the uptake of SPIO due to a reduction in the number of phagocytes [55,56]. In a study on 16 patients by Nishimura et al., results were superior with an ultrasmall SPIO-enhanced MRI with sensitivity, specificity and accuracy of 100%, 95% and 96% respectively [54]. Limits to these results were the small number of patients included and the evaluation of differences between positive and negative lymph node groups rather than node-positive versus node-negative patients [54]. Later, a feasibility study on nine patients with EC and preoperative positive lymph node status, showed that ultrasmall SPIO-enhanced MRI could identify the majority of these suspected lymph node metastases [57]. In 2009, a study on 24 consecutive patients with EC showed that whole body DWI with background body signal suppression did not result in major diagnostic improvements for N-staging [58]. Recent evidence from a 2020 study on 76 patients suggested otherwise, with DWI showing higher sensitivity than ^18^F-FDG-PET/CT for the diagnosis of metastatic lymph node in ESCC [59]. In 35 patients with EC, ECG-triggered 1.5 T MRI with turbo spin-echo (TSE) and fast short tau inversion recovery (STIR) fat suppression yielded 81% sensitivity and 98% specificity for the diagnosis of lymph node involvement [60]. As mentioned previously, in the study by Wu et al., GTV assessed on T2-weighted imaging, contrast-enhanced T1-weighted and DWI in 60 patients with ESCC was associated with the presence of lymph node metastases [40]. Another study in 46 patients with EAC found similar results [61]. Recently, a radiomic signature with nine MRI features developed in a training cohort of 90 patients and confirmed in a validation cohort of 90 patients, showed good discrimination between metastatic and non-metastatic lymph nodes [62]. As for T-staging, one study suggested that whole-tumor histogram analysis of some pharmacokinetic parameters from dynamic contrast-enhanced MRI might be able to predict regional lymph node metastases in ESCC [41].

Performance of MRI for lymph node assessment was also evaluated in the recently published meta-analysis by Lee et al. [35]. With 10 trials published between 2007 and 2019 addressing the question of differentiation of N0 disease from N1 and more advanced disease, MRI had a pooled sensitivity and specificity of 71% (95% CI: 60–80) and 72% (95% CI: 64–79) respectively [35].

Again, here, improvement of MRI modalities over the years has positively influenced its diagnostic performance for N-staging in EC. Even if recent evidence is also in favor of good sensitivity and specificity of MRI for N-staging, it remains difficult to draw firm conclusions due to the heterogeneity of MRI techniques used in the different existing studies and their small sample sizes.

### 3.3. Metastases (M) Staging

Assessment of distant metastases in EC is currently done with CT and ^18^F-FDG-PET/CT. As previously mentioned, a 2004 meta-analysis evaluating the performance of ^18^F-FDG-PET/CT for the diagnosis of distant metastases found a pooled sensitivity and specificity of 67% (95% CI: 58–76) and 97% (95% CI: 90–100), respectively [26]. In the 2008 meta-analysis by Van Vliet at al., sensitivities and specificities for the diagnosis of distant metastases were 71% (95% CI: 62–79) and 93% (95% CI: 89–97) for ^18^F-FDG-PET/CT, 52% (95% CI: 33–71) and 91% (95% CI: 86–96) for CT [46]. In one study, restaging with ^18^F-FDG-PET/CT after neoadjuvant treatment of EC resulted in the diagnosis of metastases in 8% of patients, but also with false positive findings in 5% of patients [63]. Indeed, ^18^F-FDG-PET/CT shows several limitations that can affect its performance, such as the unspecific uptake of FDG, or the existence of low uptake tumors. Furthermore, ^18^F-FDG-PET/CT is an expensive and irradiating technique. For all these reasons, the used of ^18^F-FDG-PET/CT in EC is limited to the initial workup of ESCC, and this imaging modality is rarely used for the dynamic monitoring of tumors, CT being the preferred investigation.

There is little data on the role of MRI in initial M-staging for EC. Two studies have evaluated the performance of whole-body MRI in that setting, one specifically in EC, and a second in a population with mixed gastrointestinal cancers including EC [64,65]. Compared with ^18^F-FDG-PET/CT, whole-body MRI had similar accuracy in detecting the primary tumor and lymph node metastases, and for excluding systemic metastatic disease [65]. In 49 patients with EC, both imaging modalities were able to identify distant metastases in two patients [65]. To date, there is however not enough data to recommend the routine use of whole-body MRI for M-staging in EC.

### 3.4. Target Volume Delineation before Irradiation

Accurate tumor delineation before radiotherapy, including accurate GTV, is important to ensure adequate target coverage while limiting toxicity for surrounding organs at risks. MRI is already used for tumor delineation before chemoradiotherapy in various tumor sites. Currently, delineation of EC GTV is mainly based on the combined use of CT and ^18^F-FDG-PET/CT. Nevertheless, studies suggest that the correlation between tumor length assessment by CT and pathology is weak with a frequent overestimation of this measurement by CT [66,67]. EUS has been proposed for evaluation of the longitudinal extent of the tumor, but results are difficult to translate in the radiotherapy planning process [68]. The excellent soft-tissue contrast of MRI could also substantially help increase the accuracy of tumor delineation in this setting. One study evaluated GTV delineation by 10 observers in six EC patients with MRI compared with ^18^F-FDG-PET/CT [69]. The GTV appeared smaller on breath hold T2-weighted and DWI compared to ^18^F-FDG-PET/CT acquired during free-breathing, and the main variation was seen in the cranial caudal direction [69]. Combined DWI and T2-weighted MRI sequences in two tumors of the gastrointestinal junction reduced decreased caudal border variation [69]. However, MRI delineation did not reduce interobserver variability in this study, which could partly be explained by a lack of experience of contouring GTV in EC with this imaging modality. In another study including 42 patients with operated ESCC, DWI was the more accurate modality for the measurement of GTV compared with CT and T2-weighted MRI [66]. The difference in tumor length between CT and pathology was 3.6 mm, while the difference in length between DWI and pathology was as low as 0.5 mm [66].

Finally, MRI-guided radiotherapy for EC remains under development but seems a promising option for the future. The combination of improved GTV delineation, respiratory gating, and online adaptive planning from daily non-irradiating MRI, could allow for tighter target coverage while sparing the adjacent normal organs [70].

## 4. MRI in Assessment of Treatment Response and Prediction of Recurrence

Several studies have shown improved overall survival with neoadjuvant treatment in patients with localized EC compared with upfront surgery [71,72,73]. Nevertheless, after neoadjuvant treatment and surgery, about one third of patients with EC show complete pathologic response and are possibly being unnecessarily exposed to the risks of esophagectomy [73]. Moreover, some patients will not respond to neoadjuvant treatment but will be exposed to its side effects. Overall, there is a double challenge of early identification of non-responders to neoadjuvant treatment and precise restaging after chemoradiotherapy to detect residual disease.

Previous studies and meta-analysis have shown that EUS, CT and ^18^F-FDG-PET/CT are not always adequate for the detection of residual disease in EC after neoadjuvant treatment, with poor overall accuracies [34,74,75,76]. A meta-analysis evaluating pathologic complete response after neoadjuvant treatment with various imaging techniques found pooled sensitivities of 35% (95% CI: 16–60), 62% (95% CI: 50–73), 1% (95% CI: 0–92), and 80% (95% CI: 46–95), and pooled specificities of 83% (95% CI: 71–91), 73% (95% CI: 64–81), 99% (95% CI: 81–100), and 83% (95% CI: 65–93) for CT, ^18^F-FDG-PET/CT, EUS, and MRI respectively [77]. Indeed, three-dimensional-CT volumetry evolution is not associated with histopathological tumor response [78]. EUS is limited by the difficulty of differentiating residual tumor from inflammation and fibrosis [79,80]. Finally, non-specific glucose uptake after inflammation, or the existence of low FDG uptake cancers limits the role of ^18^F-FDG-PET/CT in treatment response monitoring.

DWI and the derived apparent diffusion coefficient (ADC) can help assess tumoral metabolic activity and have shown promising results for response prediction in EC in various studies [81,82,83,84,85]. Indeed, results suggest that changes observed between baseline diffusion-weighted images and interim diffusion-weighted images (during treatment) are good prognostic and predictive biomarkers. The relative change in ADC during the first two weeks of chemoradiotherapy appears to be the most predictive for the detection of residual cancer, with a sensitivity of 100% and specificity of 75% [83]. Moreover, various b values for ADC have been evaluated in the different available studies overtime [86]. One study has suggested promising results for the prediction of treatment response in EC with the use of intravoxel incoherent motion diffusion-weighted images, which can simultaneously obtain diffusion and perfusion information from tissues without administration of a contrast agent [87]. In addition to DWI, dynamic contrast-enhanced-MRI can also be used to predict response to chemoradiotherapy in EC [88,89]. Finally, weekly T2-weighted MRI in 29 patients undergoing neoadjuvant chemoradiotherapy was able to identify volumetric changes with a significant decrease in tumor regression volume overtime [90].

In a recent meta-analysis including seven studies with EC patients treated by chemoradiotherapy, the pooled sensitivity and specificity of DWI for predicting early response to treatment were 93% (95% CI: 77–98%) and 85% (95% CI: 72–73) for the ΔADC (difference in ADC values before and after chemoradiotherapy) and 75% (95% CI: 62–84) and 90% (95% CI: 67–97) for the post ADC [91]. Even if included studies were heterogeneous with small sample sizes, these results suggest a role for DWI in the assessment of treatment response for EC.

The ongoing SANO-2 trial (NCT04886635) is currently evaluating the role of active surveillance with ^18^F-FDG-PET/CT and endoscopic biopsies after neoadjuvant chemoradiotherapy. Similarly, the randomized ESOSTRATE trial (NCT02551458) is comparing active surveillance versus surgery in patients with compete pathological response after chemoradiotherapy. Finally, the ongoing prospective study PRIDE (NCT03474341) is evaluating a multimodal prediction model including DWI and dynamic contrast-enhanced MRI to predict patient’s individual probability of complete pathological response after neoadjuvant chemoradiotherapy and identify early non-responders [92].

## 5. Discussion and Perspectives

Recent improvement in technical modalities of MRI has allowed to better assess the morphology of the normal and pathologic esophagus. Available data in EC is still scarce with small sample size studies and heterogeneity regarding clinical setting and MRI sequences and modalities. Nevertheless, currently used techniques for EC management (CT, ^18^F-FDG-PET/CT and EUS) show significant limitations, making MRI a promising tool in both initial staging (T-staging and N-staging) and assessment of response to chemoradiotherapy. There is still not enough data to conclude on the potential role of MRI in detection of distant metastasis as well as follow-up.

High field (7 T) MRI of the esophagus, currently only studied ex vivo, demonstrates an excellent sensitivity and specificity for esophageal cancer. Most importantly, it provides a clear image of the tissue layers of the esophageal wall, comparable to that of endosonography or pathology. The clinical use of this imaging modality—currently limited to the brain and the joints—for the workup of EC could allow an accurate noninvasive tumor staging, even distinguishing shallow T1 lesions potentially amenable to endoscopic resection from T1 lesions with deep submucosal infiltration or T2 lesions requiring surgical resection. In addition, its ability to discriminate fibrosis from neoplastic tissue makes of high-field MRI a promising candidate to better assess tumor response to neoadjuvant therapy.

## 6. Conclusions

In EC, combinations of different diagnostic modalities might be the way to go for optimal individual staging. In a study on 19 patients with resectable EC, PET-MRI demonstrated acceptable accuracy for T-staging compared with EUS and, although not statistically significant, higher accuracy than EUS and ^18^F-FDG-PET/CT for prediction of N-staging [93]. Furthermore, radiomics and the use of various artificial intelligence-based systems using CT imaging have recently shown promising results in a variety of diseases, including the diagnosis and monitoring of EC [94,95,96]. It may be assumed that radiomics and artificial intelligence-centered studies in EC will probably consider data from MRI for even higher performance and accuracy.

## Figures and Tables

**Table 1:** How we do it in our center: different magnetic resonance imaging sequence parameters at 1.5 T (Siemens Aera, vb20a).

Sequence Parameter	T2-Single Shot TSE	Steady States	Diffusion (EPI)	T1-Weighted before and after Gadolinium Chelate
Plane	Axial and coronal	Oblique	Axial	Axial and coronal
TE/TR (ms)	93/100	1.71/433	80/7900	2.19/4.85
Flip angle (°)	150	60	90	10
FOV (mm)	450 × 450	360 × 360	420 × 380	380 × 308
Matrix size	384 × 269	256 × 256	200 × 200	320 × 240
Slice thickness (mm)	6	10	7	2
Voxel size (mm^3^)	1.2 × 1.2 × 6	0.7 × 0.7 × 10	1.1 × 1.1 × 7	1.2 × 1.2 × 2
Number of slices	23	1	40	80
Inter-slices gap (%)	30	NA	20	20

TSE: turbo spin echo, EPI: echo planar imaging, TE: echo time, TR: repetition time, FOV: field of view, NA: not applicable.
